# Static Hyperspectral Fluorescence Imaging of Viscous Materials Based on a Linear Variable Filter Spectrometer

**DOI:** 10.3390/s130912687

**Published:** 2013-09-23

**Authors:** Patrik J. Murr, Michael Schardt, Alexander W. Koch

**Affiliations:** Institute for Measurement Systems and Sensor Technology, Technische Universität München, Theresienstrasse 90/N5, Munich 80333, Germany; E-Mails: m.schardt@tum.de (M.S.); a.w.koch@tum.de (A.W.K.)

**Keywords:** fluorescence, hyperspectral imaging, linear variable filter, CMOS 2D sensor array

## Abstract

This paper presents a low-cost hyperspectral measurement setup in a new application based on fluorescence detection in the visible (Vis) wavelength range. The aim of the setup is to take hyperspectral fluorescence images of viscous materials. Based on these images, fluorescent and non-fluorescent impurities in the viscous materials can be detected. For the illumination of the measurement object, a narrow-band high-power light-emitting diode (LED) with a center wavelength of 370 nm was used. The low-cost acquisition unit for the imaging consists of a linear variable filter (LVF) and a complementary metal oxide semiconductor (CMOS) 2D sensor array. The translucent wavelength range of the LVF is from 400 nm to 700 nm. For the confirmation of the concept, static measurements of fluorescent viscous materials with a non-fluorescent impurity have been performed and analyzed. With the presented setup, measurement surfaces in the micrometer range can be provided. The measureable minimum particle size of the impurities is in the nanometer range. The recording rate for the measurements depends on the exposure time of the used CMOS 2D sensor array and has been found to be in the microsecond range.

## 1. Introduction

Today, fluorescence measurements are established in various applications and have a broad spectrum of functionality. The main focus of current fluorescence setups can be found in the fields of biology, food technology and medicine. The quality control of fruits, for example, is realized with fluorescence measurements [1,2]. Most of these fluorescence measurement systems are based on image scanning with grayscale resolution without a separation of wavelengths. Damages and variances at the measurement surface can be detected in recorded images, due to the grayscale resolution. Other measurements with fluorescence in reflection are developed for the detection of skin cancer and are currently tested on animals, like mice or chickens [3,4]. The use of such measurement setups has some drawbacks. One of them is that at the moment, only solid and liquid materials can be investigated. Another drawback is the low scanning speed of the currently available measurement setups. A further problem is the integration of the measurement setups into a running operation. Actually, this is not possible with low effort. In addition, the current measurement setups are very expensive and massive. Thus, at the moment, there is no opportunity to get the information of a fast moving object or to integrate the setups into an established process. Yet, there are some approaches for small and cheap fluorescence spectrometers. These spectrometers, for example, consist of a combination of a linear variable filter (LVF) and a complementary metal oxide semiconductor (CMOS) 2D sensor array [5,6].

In this paper, a small and low-cost setup for fluorescence measuring of hyperspectral images with the option for moving measurements in a new application is presented. This concept is based on the method of hyperspectral imaging [7,8]. There are three well-known implementation possibilities: push-broom scan, optical bandpass filter and Fourier transform spectroscopy. For the presented setup, the push-broom method that analyzes one image per line was used. This method is currently established in aerospace applications and in scanning environmental pollution [9,10]. An advantage of this method is that spectral information corresponding to the location may be obtained. The evaluation of the measurement setup was made with viscous materials. So far, no approaches for fluorescence measurements with viscous materials have been published. At the moment, quality controls for viscous materials are realized by controlling the acoustic sounds or signals [11]. This method takes a lot of time and is very expensive, because in most of the cases, the evaluation is realized in laboratories and not directly in the production process or application site. In addition, this method does not enable continuous and contactless measurements. The consequence is that only random samples can be evaluated, and no statement for the complete product during a process can be made.

Hence, for this application, a small, low-cost and universally-usable measurement setup with illumination in the ultraviolet (UV) range was developed. The recorded images show hyperspectral fluorescence spectra in a desired wavelength range of a measurement surface of a viscous material. With this setup, a separation between the location and the wavelength in the recorded image is possible. Furthermore, a relationship between the measurement surface and the corresponding spectra in the image can be established. The consequence is that fluorescent and non-fluorescent impurities in the viscous material can be located and evaluated. Further, the quality of the viscous materials can be validated. In addition, the setup is arranged and constructed for a later integration into moving production processes.

## 2. Materials and Methods

This section covers the fundamentals of the measurement system presented in this work. Firstly, the idea will be explained, followed by the measurement setup, system characterization, calibration and analysis.

### 2.1. Idea

**Figure 1.**
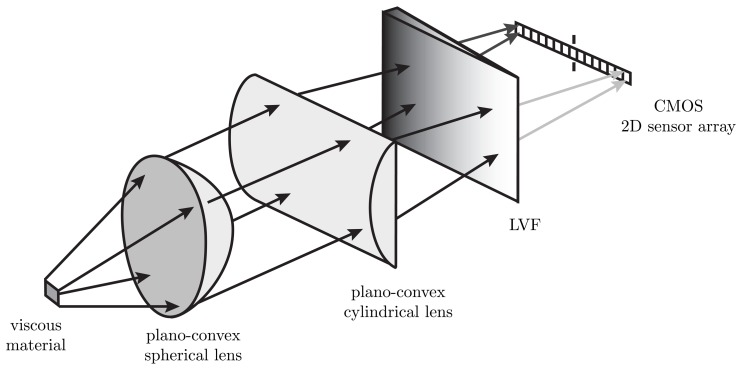
Representation of the described idea. The UV light source illuminates the viscous material (not in the figure). The resulting fluorescence of the measurement surface is denoted at an exemplary location. In addition, the spectral fluorescence imaging of the viscous material is denoted for an exemplary line array on the complementary metal oxide semiconductor (CMOS) 2D sensor array. The hyperspectral spectrometer, consisting of a linear variable filter (LVF) and a CMOS 2D sensor array, interrogates the transmitted intensity synchronously to the illumination duration of the measurement surface.

The setup of an LVF spectrometer is a special type of an optical acquisition unit. Compared to a grating spectrometer, LVF spectrometers feature several differences. LVFs have a huge aperture at high transmissivity. In a hyperspectral configuration, the dispersive element is mounted directly on the top of a CMOS 2D sensor array. Thereby, an optical hyperspectral spectrometer consisting of an LVF and a CMOS 2D sensor array is set up. This low-cost combination is the essential advantage for use as an optical adjustment unit for static fluorescence measurements on viscous materials. Figure 1 describes the idea of the developed method. The setup is explained in Section 2.2. As described in Section 1, similar methods are known from satellite-based remote sensing in the application of the push-broom principle.

In our setup, a fluorescent viscous material was provided in a notch with a constant thickness of 200 μm. A UV light source illuminates the measurement object. The fluorescence is captured by the LVF/CMOS configuration. In order to give spectral information to the regarded measurement surface, an adjustment of the lenses is used. Due to the spectral characteristic of the detection system, the spectra of a fluorescent measurement object over the location can be obtained. In addition, the existence and location of fluorescent impurities with their corresponding wavelength range can be proven. Further, non-fluorescent impurities can be measured through the decrease of the resulting fluorescence spectra.

The readout of all 1,280 × 1,024 CMOS 2D sensor array elements was synchronized with the illumination duration of a high power UV-LED. An external hardware trigger signal starts the measurement at a manually defined time. If the trigger signal is changing from a high (5 V) to a low (0 V) level, an image with the LVF/CMOS configuration is made. The duration of the recording depends on the adjusted exposure time of the CMOS 2D sensor array. After a successful data acquisition, several spectra of different points on the measurement surfaces are recorded in an image. Each hardware trigger leads to a new spectral capture of the fluorescent viscous material. The recorded image has a complete spectrum in the visible (Vis) wavelength range in each row. Each column represents a narrow-band wavelength range. The wavelength range occurs by the characteristics of LVF, which is in front of the CMOS 2D sensor array. All values in a recorded image are intensity values and are illustrated in an eight-bit gray resolution. The hyperspectral imaging is realized by the spatial resolution over the rows and the wavelength resolution over the columns on the CMOS 2D sensor array. The spot size of the measurement surface on the CMOS 2D sensor array depends on the adjudication of the lenses used in the setup. To find the best lens adjudication for the measurement setup, a simulation with the optical simulation tool Zemax was done before. In the simulation, the spot size was changed by variations of the position of the used plan-convex spherical lens. The criterion for the final lens adjudication was that all rays are still collimated as best as possible for the largest possible measurement area.

Compared to grating spectrometers, hyperspectral sampling can be realized very fast; thus, in combination with the huge aperture, it leads to an enormously reduced measurement time. In relation to the currently available hyperspectral imaging systems, this setup features higher efficiency and little adjustment effort at a low price. Due to the wafer scale production of the LVF and CMOS 2D sensor array, an enormous cost reduction is achieved. The adjustment effort is drastically reduced because of the solid-state nature of the spectral apparatus. In addition, this measurement setup is useable for other applications in which measurement objects are fluorescent. The replacement of optical components, like optical filters and light sources, is very simple, because all components in the setup are constructed modularly.

### 2.2. Measurement Setup

**Figure 2.**
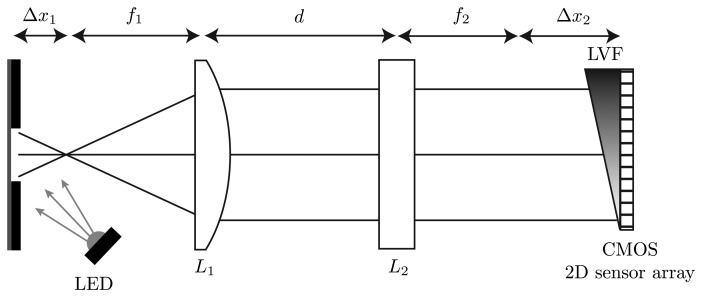
Illustration of the proposed hyperspectral fluorescence spectrometer based on an LVF/CMOS 2D sensor array configuration. In the top view of the measurement setup, the ray path of the fluorescence with the spectral information is shown. The lens adjustment consisting of a plano-convex spherical lens and a plano-convex cylindrical lens enlarges the information of the fluorescent measurement object over the complete CMOS 2D sensor array. Due to the assembly of the plano-convex cylindrical lens, this dimension contains the spectral information to a corresponding location of a considered measurement object.

**Figure 3.**
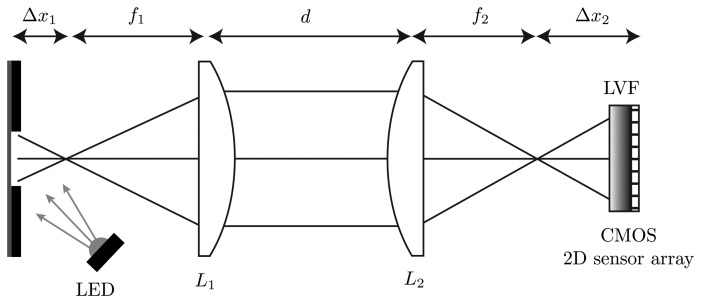
Illustration of the proposed hyperspectral fluorescence spectrometer based on an LVF/CMOS 2D sensor array configuration. In the side view of the measurement setup, the ray path of the fluorescence with the spatial information is shown. The lens adjustment consisting of a plano-convex spherical lens and a plano-convex cylindrical lens images the fluorescent measurement object onto the CMOS 2D sensor array. Due to the imaging through the plano-convex cylindrical lens, this dimension contains the spatial information of the considered measurement object.

A high-power UV-LED is used to illuminate the measurement object at an angle of 45°. The measurement object in this case is a fluorescent viscous material. The light-emitting spot on the measurement object is limited by a 750 μm slit. The resulting fluorescence is nearly collimated by the plano-convex spherical lens, *L*_1_, with the focal length *f*_1_ = 20 mm. Through a slight shift out of the focal point by Δ*x*_1_ = 1 mm, an imaging of an area on the measurement object is possible. A plano-convex cylindrical lens, *L*_2_, with the focal length *f_2_* = 25 mm is placed at the distance *d* = 30 mm. The curved side of this lens is orientated in the same direction as the slit above the measurement object. In this dimension, the plano-convex cylindrical lens images the parallel parts to the direction of the slit onto the CMOS 2D sensor array. Due to the characteristic of this lens, there is no effect of the light in the other dimension. Thus, all light rays of one point on the measurement surface are distributed in this dimension. The material of the two used lenses is borosilicate glass (BK7). The optical acquisition unit consists of an LVF (wavelength range: 400 nm–700 nm) mounted atop a CMOS 2D sensor array and is orientated in the direction of the slit atop the viscous material. The used CMOS 2D sensor array features 1,024 × 1,280 pixels with a sensitive area of 6.66 mm × 5.32 mm and has a parallel read-out capability. The size of one pixel on the CMOS 2D sensor array is 5.2 μm × 5.2 μm. The analog digital converter of the sensor has a resolution of eight bits and the exposure time can be chosen between 35 μs and 980 ms. The complete measurement setup in the plan and side view is shown in Figures 2 and 3.

### 2.3. System Characterization

To adjust the measurement setup, a resolution measurement of the LVF and a measurement of the pixel linearity on the CMOS 2D sensor array had to be carried out. The resolution of the LVF was analyzed in a previous work [12]. Considering the resolution of the LVF and the pixel size of the CMOS 2D sensor array, the system resolution is 1.6% of the LVF center wavelength. Thus, the measurement setup can measure impurities that are larger than ten nanometers. The pixel linearity of the CMOS 2D sensor array was determined with a light-emitting film in the visible wavelength range, which illuminated the complete CMOS 2D sensor array with a constant intensity. This measurement was done with and without the LVF atop the CMOS 2D sensor array. The intensity of the illumination was adjusted by two polarization filters in constant rotation steps of 5°. For each measurement step, the maximum variance of the mean pixel intensity over all pixels was lower than 3.1%. Furthermore, for each pixel, the maximum variance from the ideal linearity over all intensity values was lower than 1.4%. Due to the results of the measurement characterization, it has to be stated that all pixels on the CMOS 2D sensor array show nearly linear characteristics. In addition, the signal-to-noise ratio (SNR) of the measurement setup was calculated as 59.

### 2.4. Calibration and Measurement Process

A wavelength calibration of the complete measurement setup is necessary for an interpretation and evaluation of the fluorescence data. The wavelength range is defined by the used LVF and CMOS 2D sensor array. For the wavelength calibration, a mercury lamp was used as the illumination, emitting four distinctive peaks in the range of the LVF. The four peaks are located at 406 nm, 436 nm, 546 nm, and 578 nm. The detected wavelengths of each pixel on the CMOS 2D sensor array are filtered by the LVF and change with little shifting of the position of the LVF. Hence, the specific combination of the LVF and CMOS 2D sensor array has to be calibrated with respect to its wavelength distribution. Because of the linear wavelength characteristic of the LVF, each row of the CMOS 2D sensor array was calibrated by applying a linear fit algorithm to the measured wavelengths of the characteristic peaks. The wavelength calibration method is described in more detail in [13]. After a successful wavelength calibration, it is possible to allocate for each intensity value the corresponding wavelength, and the LVF/CMOS configuration can be integrated into the measurement setup. In addition, it is possible to create a complete spectrum for each row of the CMOS 2D sensor array. Due to the eight-bit intensity resolution of the CMOS 2D sensor array, there is no additional intensity calibration for the system necessary.

A measurement process of the fluorescence spectra consists of four phases. First, the parameters for the illumination duration of the UV-LED, the frequency for the external hardware trigger, the exposure time of the CMOS 2D sensor array and the periodic time of one measurement have to be set. These settings are necessary, so that the images have no overexposure and the fluorescence intensity does not decrease through a warm-up of the viscous material. In the second phase, the data acquisition starts by the manually defined hardware trigger. Then, data processing is deployed with respect to the recorded measurement signals. Finally, an analysis and interpretation of the resulting data is realized.

In summary, the described measurement setup is a hyperspectral fluorescence spectrometer where the spatial information along the direction of the slit is detected in one dimension of the CMOS 2D sensor array. The other dimension of the CMOS 2D sensor array shows the corresponding spectral information divided by the LVF.

### 2.5. Analysis

Due to the different concentrations and properties of the fluorescent components of the viscous materials, it is important that no fluorescence decreasing effects during the measurements exist. One of these effects occurs especially at static measurements, where the measurement surface is illuminated for an extended time and warm-up. Thereby, the molecules in the viscous materials obtain stronger vibrations, and the collision probability increases with rising object temperature. This is known as the quenching effect and leads to a decrease of the fluorescence signal [14]. For our case, the effect can be classified at the dynamic fluorescence extinction and can be described with the Stern–Volmer Equation [15]. This equation can be written as:

(1) $$\frac{F_{0}}{F}=1+K_{D}[Q]$$

where *F*_0_ and *F* are the fluorescence intensities in the absence and presence of the quencher and *Q* is the concentration of the quencher. The dependency of the temperature in the equation is represented by the Stern-Volmer constant, *K_D_*. The value of *K_D_* is inversely proportional to the temperature of the viscous fluorescent materials.

The root-mean-square error (RMSE) between a reference and measured fluorescence spectrum gives the difference of both in the quantity being measured (in this case, % fluorescence) and can be written as:

(2) $$\text{RMSE}(j)=\sqrt{\frac{\Sigma_{i=1}^{n}{\left(F_{i,\text{ref}}-F_{i,j}\right)}^{2}}{n}}$$

where *n* is the number of sampling points of the spectrum and *j* is the row number of the CMOS 2D sensor array with a complete spectrum.

## 3. Results and Discussion

In this section, exemplarily static fluorescence measurements are presented, and a discussion of the obtained results is carried out. The efficiency of the complete system is demonstrated by fluorescence measurements with and without impurities in a viscous material.

**Figure 4.**
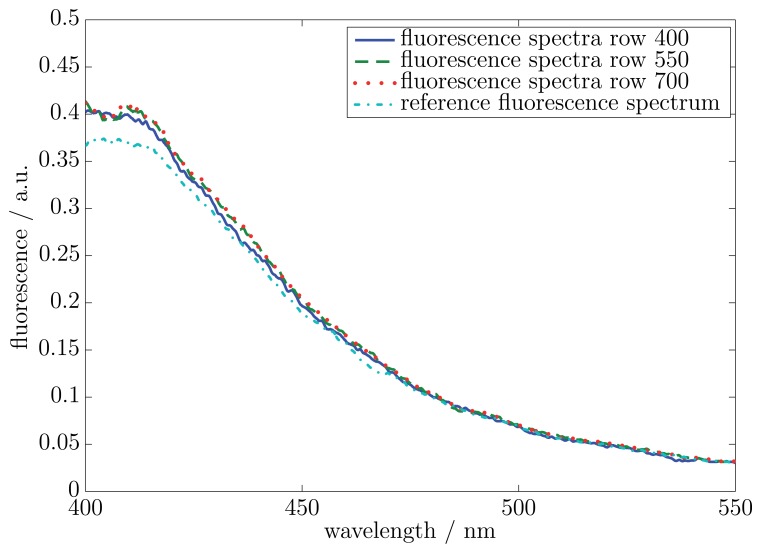
Static measured hyperspectral fluorescence spectra of a viscous material without an impurity. Exemplarily, spectra of rows 400, 550 and 700 and a reference spectrum of a UV-Vis-spectrometer are illustrated. The location of the three rows is on both sides and in the middle of the relevant area on the CMOS 2D sensor array. The intensities of all fluorescence spectra are specified in arbitrary units (a.u.).

In the first measurement, a fluorescent viscous material with no impurity was used. The measurement surface was illuminated with the wavelength of 370 nm (10 nm full width at half maximum, FWHM). The resulting fluorescence spectra included a wavelength range from 380 nm to 550 nm. The sample thickness was in all places 200 μm. A reference measurement was carried out by a commercial UV-Vis-spectrometer. The measured spectra started at a wavelength of 400 nm. Due to the optical properties of the LVF, the wavelength was restricted on a range of 400 nm to 700 nm. Figure 4 shows the results of the measured fluorescence spectra for three exemplary rows on the CMOS 2D sensor array. The locations of the three rows are on both sides and in the middle of the relevant area on the CMOS 2D sensor array. The regarded area on the CMOS 2D sensor array for the hyperspectral imaging was restricted through the dimensions of the LVF. The relevant area comprised row 400 to 700 and all columns (6.66 mm × 1.56 mm). Furthermore, Figure 4 illustrates a reference fluorescence spectrum of a UV-Vis-spectrometer.

**Figure 5.**
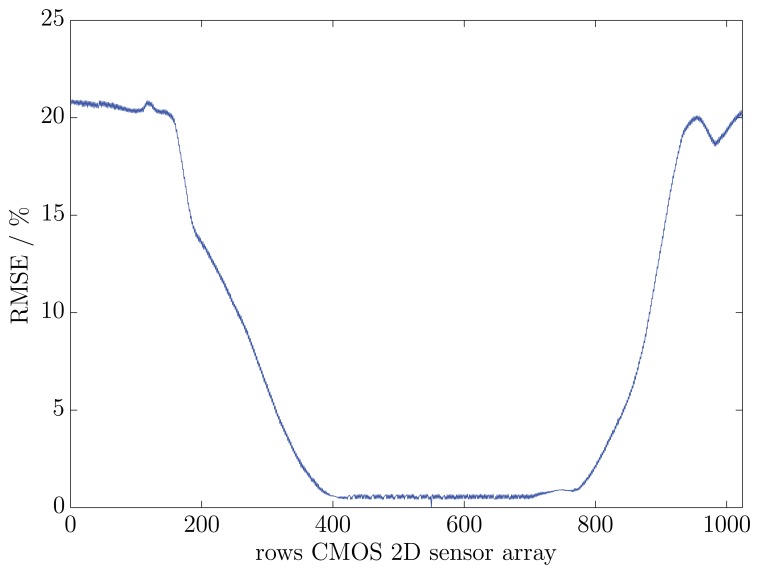
Root-mean-square error (RMSE) trend between a reference row and all other rows of the CMOS 2D sensor array. The chosen rows (row number 400 to 700) show a maximal RMSE of 0.65% to the reference row (row number: 442).

The results of the three exemplary fluorescence spectra show a good accordance between one another. The reference spectrum of the UV-Vis-spectrometer is almost equivalent to the three exemplary rows. The variances between the reference spectrum and the exemplary spectra were calculated with Equation (2). The differences amount to 1.75% (row number: 400), 1.69% (row number: 550) and 1.42% (row number: 700). In order to prove that all rows on the CMOS 2D sensor array have the same fluorescence spectrum, a RMSE trend was calculated. As a reference spectrum, the middle row (row number: 550) on the CMOS 2D sensor array was chosen. In Figure 5, the RMSE trend of all other relevant rows to the reference row is illustrated.

The results agree well with the presumption that the measured viscous material contains no impurities, which leads to a constant fluorescence over the complete measurement surface. The highest RMSE of all relevant rows is 0.65% at row 442. The average RMSE of the considered rows is 0.55%. Minor differences in the measured spectra compared to the reference row can be explained with small irregularities in the used optical components.

**Figure 6.**
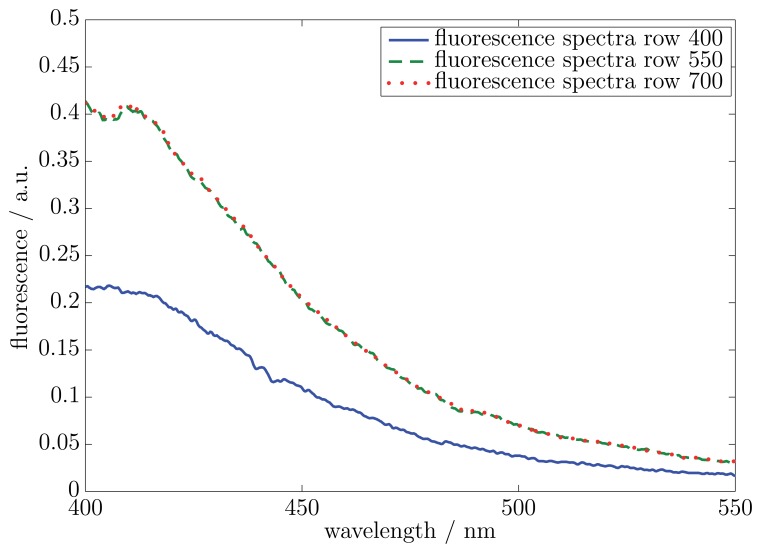
Static measured hyperspectral fluorescence spectra of a viscous material with a non-fluorescent impurity. Exemplary fluorescence spectra of rows 400, 550 and 700. The non-fluorescent impurity is in the area of row 400 on the CMOS 2D sensor array.

After the successful proof of the idea presented in Section 2.1 by references to fluorescent viscous materials, another measurement with a non-fluorescent impurity at a known location was performed. In this measurement, the same area on the CMOS 2D sensor array as in the previous measurement was considered. In Figure 6, the fluorescence spectra of three exemplary rows are shown. The locations of the exemplary rows correspond to the investigated rows of the first measurement.

The results in Figure 6 demonstrate two identical fluorescence spectra and a fluorescence spectrum with a lower intensity. This confirms that in the area of row 400, a non-fluorescent impurity is included, thus enabling the measurement and location of non-fluorescent impurities in viscous materials based on the hyperspectral imaging with a small and low-cost LVF-spectrometer.

Besides measurements of a non-fluorescent impurity in viscous materials, it is possible to use the system for measurements in viscous materials with a fluorescent impurity. The expected results of the spectra for the area without an impurity should be similar to the spectra in Figure 4. For the area with the fluorescent impurity in the viscous material, an increase in the fluorescence intensity over the complete wavelength range is expected. This is different from the non-fluorescent impurity, where a decrease of the fluorescence intensity over the complete spectrum exists.

A first step in order to increase the robustness and accuracy of the system is to integrate the LVF directly atop the CMOS 2D sensor array. Further, it is desirable to increase the area of the CMOS 2D sensor array, which is covered with an LVF. Moreover, measurements with higher resolutions of the CMOS 2D sensor array up to twelve bits can be tested. In addition, the setup can be modified for moving measurements of viscous materials or other applications.

Other possibilities are measuring different viscous materials and investigating different impurities in one sample. An aim of such a measurement can be, e.g., the identification and location of several fluorescent impurities in one sample by variations in the fluorescence spectra.

## 4. Conclusions

A new approach and application for static hyperspectral fluorescence measurements has been presented. The main idea is to build a universally useable, contactless, online, small and low-cost LVF-spectrometer. The acquisition unit with lens adjustment is mounted vertically above the sample. The light source illuminates the viscous material at an angle of 45° at a wavelength of 370 nm. By an appropriate synchronization between the illumination time and the exposure time of the CMOS 2D sensor array, it is possible to get static fluorescence spectra of a measurement surface separated in location and wavelength.

Exemplary static measurements of a fluorescent viscous material with and without a non-fluorescent impurity show the efficiency of this approach. A good agreement with a reference fluorescence spectrum of a UV-Vis-spectrometer was achieved. In addition, the accordance between the relevant fluorescence spectra at the CMOS 2D sensor array in a non-fouled fluorescent sample was illustrated. The possibility to detect and locate a non-fluorescent impurity on a measurement surface of a viscous material through the fluorescence spectra has been proven. Actually, the minimum cross-section dimension for a successful detection of an impurity in the measurement system is about 75 nm. The maximum size for the impurity is limited through the dimension of the slit in the system.

The implementation of this approach for moving measurements of fluorescent samples is conceivable due to small changes in the presented setup and software. The described technology enables a cost-effective and high-speed monitoring of the production processes of viscous materials based on fluorescence. Such an application has not been developed until now.

By replacing the optical components, like LVF and the light source, with identically constructed components with other optical parameters, it is possible to use this setup in other applications where the measurement objects are fluorescent. Further investigations will show the suitability of this setup for the detection and location of different kinds of impurities in fluorescent viscous materials. In addition, the efficiency of the approach for moving fluorescence measurements and other applications will be determined.
